# A head-to-head comparison of high-frequency ultrasound and magnetic resonance imaging for preoperative assessment of ATFL tears and concomitant CFL injuries

**DOI:** 10.3389/fsurg.2026.1863972

**Published:** 2026-07-29

**Authors:** Binbin Tang, Lei Wang, Yuanjun Zheng, Shu Gong, Yongmei Wang

**Affiliations:** 1 Department of Cardiovascular Ultrasound, The Second Qilu Hospital of Shandong University, Jinan, Shandong, China; 2 Department of Emergency, The Second Qilu Hospital of Shandong University, Jinan, Shandong, China

**Keywords:** anterior talofibular ligament (ATFL), diagnostic accuracy, high-frequency ultrasound(HFUS), lateral ankle ligament injury, magnetic resonance imaging (MRI)

## Abstract

**Background:**

This study aims to comparatively evaluate the diagnostic performance of high-frequency ultrasound (HFUS) and magnetic resonance imaging (MRI) in the assessment of anterior talofibular ligament (ATFL) injuries, particularly in the context of associated concomitant injuries. By analyzing sensitivity, specificity, accuracy, and inter-modality agreement, we seek to determine the relative clinical utility of these two imaging modalities for comprehensive evaluation of lateral ankle instability and multi-structure involvement.

**Methods:**

A retrospective, single-center study was conducted on clinical data from 123 surgically treated patients suspected of ATFL ankle injuries who presented to our hospital between May 2015 and June 2025. All patients underwent both HFUS and MRI examinations prior to surgery. Surgical findings were used as the reference standard to compare the diagnostic performance of HFUS and MRI in assessing the severity of acute ATFL injuries. The diagnostic efficacy of HFUS and MRI for ATFL injuries was evaluated using receiver operating characteristic (ROC) curve analysis.

**Results:**

In grading ATFL injuries, HFUS demonstrated significantly higher diagnostic accuracy compared to MRI (90.24% vs. 78.05%, *p* < 0.01). The AUC for detecting complete ATFL tears was also markedly superior for HFUS (0.938, 95% CI: 0.898–0.975) compared to MRI (0.857, 95% CI: 0.792–0.917). However, in detecting concomitant calcaneofibular ligament (CFL) injuries, MRI demonstrated substantially higher sensitivity (95.83%, 95% CI: 91.67–99.00) than HFUS (63.54%, 95% CI: 53.69–72.00), highlighting its superior performance in identifying combined ATFL-CFL injuries.

**Conclusion:**

HFUS demonstrates better diagnostic performance for preoperative grading of ATFL tear severity, whereas MRI shows moderate diagnostic capability in detecting concomitant CFL injuries in the setting of combined lateral ankle ligament trauma. These findings support a complementary imaging strategy, with HFUS as a useful initial tool for ATFL assessment and MRI reserved for comprehensive evaluation when concomitant CFL or other pathologies are suspected.

## Introduction

Acute ankle injuries most commonly involve the lateral ligament complex, with the anterior talofibular ligament (ATFL) being the most frequently injured structure (1, 2). As the weakest component of this complex, the ATFL is highly susceptible to injury and is often involved in concurrent, or composite, ligamentous and osteochondral pathologies (3, 4). Inadequate diagnosis and management of these injuries, particularly when multifaceted, can lead to chronic ankle instability, recurrent sprains, and accelerated joint degeneration (5, 6). Therefore, the accurate and timely delineation of ATFL injuries, especially within a complex injury pattern, is crucial for guiding appropriate treatment and optimizing long-term outcomes.

However, ATFL injuries frequently do not occur in isolation. Owing to anatomical vulnerability and typical injury mechanisms, the ATFL is often involved alongside damage to adjacent ligaments, especially the calcaneofibular ligament (CFL), resulting in a composite injury pattern (7, 8). Both High-Frequency Ultrasound (HFUS) and magnetic resonance imaging (MRI) are established for evaluating isolated ATFL tears (9–11). A recent systematic review and meta-analysis by Colò et al., encompassing nine studies, reported a pooled sensitivity of 96.9% (95% CI 94–99) for HFUS vs. 88.5% (95% CI 85–91) for MRI in detecting ATFL lesions (11). Seok et al. reported pooled sensitivity and specificity of ultrasound for ATFL injury of 93% and 89%, respectively (12). However, each modality carries well-recognized limitations that are particularly consequential in the clinical setting where both ATFL grading and CFL detection are required. The diagnostic performance of HFUS is operator-dependent. Zhou et al. reported that accuracy for detecting complete ATFL tears ranged from 88.5% for senior radiologists to 67.7–72.3% for junior radiologists, with poor inter-observer agreement (*κ* < 0.75) for most sonographic signs (13). MRI, while less operator-dependent, has limited specificity in chronic injuries, where scar tissue may mimic intact ligaments (14, 15). Consequently, the comparative performance of HFUS and MRI specifically for preoperative assessment—with surgical findings as the reference standard—remains unclear.

Two critical questions arise: how reliably do HFUS and MRI grade ATFL severity in composite injuries, and which modality better detects concomitant CFL tears? Therefore, this study aimed to address the following objectives: (1) to evaluate and compare the diagnostic performance of HFUS and MRI in grading ATFL injuries (e.g., partial vs. complete tears) within a composite injury context; and (2) to directly compare the diagnostic efficacy of HFUS and MRI in identifying concomitant CFL injuries. Using surgical findings as the reference standard, this study seeks to provide an evidence-based rationale for imaging modality selection in the diagnostic assessment of lateral ankle ligament complex injuries, thereby supporting optimized diagnostic pathways and clinical decision-making.

## Materials and methods

### Study population

This was a retrospective, single-center study. We consecutively identified patients who underwent surgical treatment for an anterior talofibular ligament (ATFL) injury with concomitant lateral ligamentous pathologies at our institutions between May 2015 and June 2025. Patients were included if they had complete preoperative HFUS and MRI reports and documented surgical findings. A total 123 patients (73 males, 50 females; mean age 32.56 ± 14.67 years, range 12–70 years) met the eligibility criteria and were included in the final analysis.

**Inclusion criteria were as follows**: 1) diagnosis of an acute ATFL injury (defined as <6 weeks from injury to imaging); 2) completion of preoperative high-frequency ultrasound (HFUS), magnetic resonance imaging (MRI), and surgical exploration; 3) presence of at least one of the following surgical indications: (a) imaging evidence (HFUS or MRI) of a complete (Grade III) ATFL tear combined with strong patient preference for surgical intervention; (b) concomitant distal fibular and/or talar fracture requiring surgical fixation (e.g., displaced avulsion fracture >2 mm, intra-articular fracture); (c) objective ankle instability, defined as abnormal anterior drawer and varus stress tests, with a talar tilt angle >10° compared to the contralateral side or an absolute value >15° on varus stress radiography; (d) high functional demand (e.g., competitive athletes or occupations requiring rapid return to activity); or (e) progressive pain and swelling despite 2–3 weeks of adequate immobilization and rehabilitation, suggesting an undetected avulsion or intra-articular fracture requiring surgical exploration; and 4) no prior specific treatment administered before imaging.

**Exclusion criteria were as follows**: 1) contraindications to HFUS, MRI, or surgery; 2) history of ipsilateral ankle sprain, chronic ankle pain, or instability (symptoms >6 months); 3) previous lateral ankle fracture or related surgical intervention on the affected side; and patients who improved with conservative treatment (e.g., symptom resolution after 2 weeks of non-surgical management).

### Imaging acquisition

#### Ultrasound

All patients underwent ankle ultrasound examination with the patient seated and the foot in full plantar flexion. Examinations were performed by musculoskeletal radiologists with at least 5 years of experience following a standardized scanning protocol using high-frequency linear-array probes (L12-5) on PHILIPS EPIQ systems (EPIQ-7C and EPIQ-CVx). For concomitant CFL assessment, patient positioning was adjusted as needed based on anatomical landmarks.

#### MRI

MRI was performed using a Philips Ingenia Ambition S system. Patients were placed in a supine position with the lower limb in natural extension and the ankle in a neutral position. The protocol included T1-weighted, T2-weighted, and fat-suppressed sequences acquired in orthogonal planes.

### Image interpretation

All ultrasound and MRI images were interpreted prospectively, prior to surgery. Ultrasound images were reviewed by one group of senior musculoskeletal radiologists, and MRI images were reviewed independently by a separate group. For each modality, the final classification of ATFL injuries was determined by consensus between two radiologists. Both groups were blinded to the other modality's findings and to surgical results. Basic clinical information (age, sex, injury mechanism) was available to both groups, reflecting routine clinical practice.

### Blinding

All imaging interpretations were performed prospectively, before surgery. The HFUS and MRI studies were read independently by two separate groups of musculoskeletal radiologists, who were blinded to each other's findings. Neither group had access to the surgical findings, as surgery was performed after the imaging interpretations. Both groups had access to basic clinical information (e.g., patient age, sex, and mechanism of injury), which reflects routine clinical practice. The treating surgeons, however, had access to both HFUS and MRI results for surgical planning.

### Diagnostic criteria

#### Avulsion fracture

Discontinuity of the talar or fibular attachment site of the ATFL, with visualization of a detached bone fragment that appears hyperechoic on HFUS and hypointense on MRI.

#### Calcaneofibular ligament (CFL) injury

Swelling and thickening of the ligament with loss of normal fibrillar architecture, partial or complete fiber discontinuity, and possible associated joint effusion.

#### ATFL injury classification

Ligament injuries were classified into four grades according to both imaging and intraoperative findings:

a) Normal: The ligament appears continuous, with uniform thickness, normal echogenicity/signal intensity, and preserved fibrillar architecture.
b) Sprain (Grade I): Focal ligament thickening (>2 mm) with reduced echogenicity (US) or increased T2 signal intensity (MRI), but maintained structural continuity.
c) Partial Tear (Grade II): Partial disruption of ligament fibers, resulting in focal attenuation or irregularity, often accompanied by peri-ligamentous edema and/or joint effusion.
d) Complete Tear (Grade III): Complete discontinuity of the ligament fibers with visible retraction of the torn ends, typically associated with surrounding joint effusion and/or an avulsion fracture.

### Statistical methods

All statistical analyses were conducted using R software (version 4.3.1). This is a retrospective study; all eligible patients meeting the inclusion criteria between May 2015 and June 2025 were included, and no *a priori* sample size calculation was performed. Using surgical findings as the gold standard, the diagnostic performance of HFUS and MRI for classifying ATFL injuries was evaluated in terms of accuracy, sensitivity, specificity, positive predictive value, and negative predictive value. Paired comparisons between the two modalities were performed using McNemar's test. Receiver operating characteristic (ROC) curves were generated to assess overall diagnostic accuracy. Because the imaging grades were ordinal (Normal, Grade I, Grade II, Grade III), the ROC analysis was performed by defining multiple binary thresholds (Grade ≥I, ≥II, ≥III) against the surgical reference standard. The area under the curve (AUC) was calculated with 95% confidence intervals using the nonparametric method, and the optimal diagnostic threshold was identified using the Youden index. The difference between the AUCs of HFUS and MRI was tested using DeLong's test for paired ROC curves. The inter-rater reliability between HFUS and MRI assessments was evaluated using linearly weighted kappa statistic, implemented via the irr package (version 0.84.1). Agreement was interpreted as follows: kappa values ≥0.75 indicated good agreement; values between 0.40 and 0.75 indicated moderate agreement; and values <0.40 indicated poor agreement. We employed a robust bootstrap-based approach to compare paired Kappa coefficients. Bootstrap confidence intervals were computed primarily using the bias-corrected and accelerated (BCa) method, with percentile intervals as a fallback. A *P*-value < 0.05 was considered statistically significant.

### Ethics statement

This retrospective study was approved by the institutional ethics committee with a waiver of informed consent, as it involved the analysis of existing, anonymized clinical data.

## Results

### Basic characteristics of study population

This study included 123 patients (73 males and 50 females) aged 12 to 70 years, with a mean age of 32.56 ± 14.67 years. Each patient underwent a complete diagnostic workup, including high-frequency ultrasound (HFUS), magnetic resonance imaging (MRI), and surgery. There were 65 complete anterior talofibular ligament (ATFL) tears, 36 partial ATFL tears, and 22 sprain or normal ATFLs, as confirmed by surgery (Table 1).

**Table 1:** Basic characteristics.

Characteristics	Value
Age(y)	32.56 ± 14.67
Sex	
Male	73
Female	50
Affected side	
Left	63
Right	57
Both	3
Injury mode	
Sprain	45
Trauma	78
Injury types	
Complete tear(III)	65
Partial tear(II)	36
Sprain(I)	22

Data are presented as mean ± standard deviation.

### Diagnostic performance of HFUS and MRI in grading ATFL injuries

Overall diagnostic performance for detecting any ATFL injury (i.e., grades 1–3) was first evaluated (Table 2). HFUS demonstrated an accuracy of 90.24% (111/123), a sensitivity of 90.43%, and a specificity of 95.46%. In comparison, MRI yielded an accuracy of 78.05%, a sensitivity of 81.20%, and a specificity of 90.45%. Weighted kappa values indicated excellent inter-observer agreement for HFUS (*κ* = 0.876; 95% CI: 0.808, 0.944) but moderate agreement for MRI (*κ* = 0.668; 95% CI: 0.555, 0.781). The difference between the two modalities was statistically significant (*P* < 0.001). Detailed test results for each patient are provided in Supplementary Table S1.

**Table 2:** Comparison of HFUS and MRI findings with surgical exploration in the diagnosis of ATFL injuries.

Test	Linear Weighted Kappa	*P* value	Sensitivity (%)	Specificit (%)	Accuracy
HFUS	0.876 (0.808, 0.944)	<0.001	90.43 (84.16, 95.92)	95.46 (92.77, 97.80)	90.24%
MRI	0.668 (0.555, 0.781)	<0.001	81.20 (72.79, 86.36)	90.45 (86.36, 93.98)	78.05%

Continuous data are summarized as point estimates with 95% confidence intervals. A *P* value of <0.05 was considered statistically significant. HFUS, high-frequency ultrasound; MRI, magnetic resonance imaging; ATFL, anterior talofibular ligament.

As all patients exhibited ATFL abnormalities during surgery (i.e., no true grade-0 cases), diagnostic performance was subsequently evaluated by dichotomizing injury severity: grade 3 (complete tear) was classified as positive, while grades 1–2 (partial injury or sprain) were classified as negative. When compared against intraoperative findings (Table 3), HFUS demonstrated a diagnostic accuracy of 93.5% (115/123) for ATFL grading, which was significantly higher than the 85.37% (105/123) achieved by MRI. The difference in diagnostic accuracy between HFUS and MRI was assessed using McNemar's test, which yielded a p-value of less than 0.01. Furthermore, the area under the curve (AUC) for HFUS in detecting complete ATFL tears was 0.938 (95% CI: 0.898–0.975), significantly higher than that for MRI (0.857, 95% CI: 0.792–0.917), with a difference of 0.081 (95% CI: 0.013–0.150, *p* = 0.020 by DeLong's test).

**Table 3:** Comparison of HFUS and MRI findings with surgical exploration in diagnosing complete tears associated with ATFL injuries.

Test	AUC	Linear Weighted Kappa	Sensitivity (%)	Specificity (%)	Accuracy
HFUS	0.938 (0.898, 0.975)	0.870 (0.784, 0.957), *P* < 0.001	87.69 (79.66, 95.16)	100.00	93.50%
MRI	0.857 (0.792, 0.917)	0.709 (0.585, 0.830), *P* < 0.001	80.00 (69.70, 89.29)	100.00	85.37%

Continuous data are summarized as point estimates with 95% confidence intervals. A *P* value of <0.05 was considered statistically significant. HFUS, high-frequency ultrasound; MRI, magnetic resonance imaging; ATFL, anterior talofibular ligament.

### Comparative diagnostic performance of HFUS and MRI for concomitant CFL injury

With intraoperative findings as the reference standard, MRI demonstrated significantly superior diagnostic performance compared to HFUS. The Area Under the Curve (AUC) for MRI was 0.979 (95% CI: 0.958–0.995), indicating excellent diagnostic ability, compared to 0.818 (95% CI: 0.768–0.865) for HFUS. DeLong's test confirmed a significant difference, with a difference in AUC of −0.161 (95% CI: −0.215 to −0.108, *p* < 0.001).The agreement with surgical findings, as measured by Linear Weighted Kappa, was almost perfect for MRI at 0.910 (95% CI: 0.823, 0.996; *P* < 0.001), whereas HFUS showed only fair agreement (Kappa = 0.433; 95% CI: 0.303, 0.564; *P* < 0.001). In terms of sensitivity, MRI detected 95.83% (95% CI: 91.67, 99.00) of injuries, substantially higher than the 63.54% (95% CI: 53.69, 72.00) sensitivity of HFUS. Both modalities achieved perfect specificity of 100%. Consequently, the overall accuracy of MRI was 96.75%, vastly outperforming HFUS, which had an accuracy of 71.54% (Table 4). The 100% specificity indicates that no false-positive CFL diagnoses occurred in this cohort; all cases classified as positive on either imaging modality were confirmed surgically. However, this finding should be interpreted with caution, as it may be influenced by the limited number of negative CFL cases in the study population. These results suggest that MRI provides meaningful diagnostic performance for associated calcaneofibular ligament (CFL) injuries, whereas HFUS alone is insufficient for reliable CFL assessment in this cohort.

**Table 4:** Comparison of HFUS and MRI findings with surgical exploration for diagnosing combined ATFL and CFL injuries.

Test	AUC	Linear Weighted Kappa	Sensitivity (%)	Specificity (%)	Accuracy
HFUS	0.818 (0.768–0.865)	0.433 (0.303, 0.564), *P* < 0.001	63.54 (53.69, 72.00)	100	71.54%
MRI	0.979 (0.958–0.995)	0.910 (0.823, 0.996), *P* < 0.001	95.83 (91.67, 99.00)	100	96.75%

Continuous data are summarized as point estimates with 95% confidence intervals. A *P* value of <0.05 was considered statistically significant. HFUS, high-frequency ultrasound; MRI, magnetic resonance imaging; ATFL, anterior talofibular ligament. CFL, concomitant calcaneofibular ligament.

## Discussion

The accurate preoperative delineation of anterior talofibular ligament (ATFL) injury severity, particularly in the context of concomitant lateral ligamentous damage, is critical for guiding surgical decision-making and prognostic evaluation. This study, employing intraoperative findings as the reference standard, provides a direct comparative analysis of high-frequency ultrasound (HFUS) and magnetic resonance imaging (MRI) in this specific clinical scenario. Our principal finding is that HFUS demonstrated superior diagnostic performance to MRI in grading ATFL tears within a composite injury pattern, both in terms of overall accuracy(93.5% vs. 85.37%) and discriminative ability for complete tears(AUC: 0.938 vs. 0.857).

Our results demonstrate that HFUS outperformed MRI in diagnostic sensitivity, detecting 87.69% (95% CI: 79.66–95.16) of cases, compared with MRI's sensitivity of 80.00% (95% CI: 69.70–89.29). These findings are largely consistent with previous meta-analyses, which highlight the high diagnostic accuracy of ultrasound in evaluating lateral ankle ligament injuries (12–14, 16). The advantage of HFUS becomes apparent when contrasted with the inherent limitations of MRI in this specific task. Notwithstanding its established role as the comprehensive imaging gold standard for detecting associated osseous and osteochondral pathologies (17). MRI exhibits more variable performance in precisely delineating the extent of ligamentous disruption (10, 11, 17). Static imaging, standard slice thickness, and susceptibility to artifacts (e.g., the “magic angle” effect in the obliquely oriented ATFL) are inherent limitations of MRI. These factors may contribute to over- or under-estimation of tear completeness (10, 18). These factors can be compounded in the setting of composite injuries, where altered anatomy may further confound assessment. HFUS addresses these limitations through its technical synergy with superficial ligament anatomy. First, its high resolution visualizes fiber-level details crucial for distinguishing partial from complete ruptures. Second, and critically, it enables dynamic, real-time stress examination (e.g., anterior drawer test), providing a direct assessment of functional instability that static MRI cannot replicate. The reported higher sensitivity of ultrasound when performed acutely (16, 19) further aligns with HFUS's ability to exploit the optimal acoustic contrast provided by early post-injury hematoma and edema. Thus, HFUS offers a unique combination of morphological and functional insight, justifying its enhanced accuracy for preoperative grading and planning.

Previous meta-analyses have reported pooled sensitivities of 93%–97% for HFUS and 88%–89% for MRI in detecting ATFL injuries (11, 12). Our results (HFUS 87.7% sensitivity, 93.5% accuracy; MRI 80.0% sensitivity, 85.4% accuracy) fall within the lower range of these estimates. This slight discrepancy likely reflects the more stringent reference standard used in our study compared to the clinical follow-up or non-surgical standards predominantly used in prior meta-analyses. Besides, our study revealed a distinct performance profile in diagnosing concomitant calcaneofibular ligament (CFL) injuries: MRI demonstrated superior sensitivity (95.83% vs. 63.54%), while both modalities maintained high specificity. This pattern suggests that while a positive finding on either HFUS or MRI is highly reliable for confirming a CFL tear, MRI is a more robust tool for ruling it out. The observed discrepancy in sensitivity can be primarily attributed to anatomical and technical factors (12). The CFL lies deeper than the ATFL, and its oblique course often results in suboptimal orientation relative to the ultrasound beam (20). This can lead to anisotropic artifact, mimicking hypoechoic tears or obscuring true defects, thereby reducing HFUS's detection rate. In contrast, MRI is unaffected by such angle-dependent limitations. Its multi-planar capability and superior contrast resolution for bone marrow and soft tissue edema allow for more consistent visualization of the entire CFL, enhancing its ability to detect injury.

Collectively, these findings advocate for a nuanced, modality-specific diagnostic approach. While MRI remains indispensable for ruling out associated osseous and complex injuries (e.g., bone bruises, syndesmotic involvement), its precision in grading isolated ATFL tears can be confounded in the setting of composite ligamentous damage. Conversely, HFUS excels in this specific task due to its dynamic, high-resolution assessment. Therefore, in the diagnostic workup of lateral ankle instability, HFUS may be prioritized for precise ligament characterization, while MRI is optimally reserved for comprehensive assessment when clinical suspicion of concomitant pathology is high.

The strengths of this study include the use of surgical confirmation as the reference standard, a relatively substantial sample size for a focused diagnostic study, and the blinding of radiologists to the other modality's results. However, several limitations must be acknowledged. First, this single-center with a specific surgical case mix limit generalizability to broader populations. Second, HFUS performance is operator-dependent, and our results reflect the expertise of senior musculoskeletal radiologists; the outcomes might differ in settings with less experienced operators. Third, the dichotomization of injury severity, while clinically relevant for surgical planning, simplifies the spectrum of ligament pathology. Fourth, our analysis only included patients who underwent surgery, so the findings may not generalize to non-surgical or conservatively managed populations. Fifth, interobserver reliability between radiologists was not assessed. In this retrospective study, only the final consensus interpretations (as used for clinical decision-making) were available from the medical records. Therefore, reader-reader kappa could not be calculated. Future prospective studies should pre-specify independent reads to evaluate interobserver agreement. Finally, the 10-year recruitment period may introduce minor changes in imaging hardware or protocols. Although the critical ultrasound transducer and MRI core sequences remained unchanged, subtle temporal variations cannot be fully excluded. Any such changes would likely bias results toward the null, rather than overestimating diagnostic performance.

In conclusion, this study demonstrates that in the specific setting of composite lateral ankle ligament injuries requiring surgical assessment, HFUS provides more accurate grading of ATFL tear severity compared to conventional MRI. These findings suggest that HFUS can complement MRI in the preoperative workup of these patients, particularly when precise characterization of the ATFL is paramount. Future prospective, multi-center studies evaluating standardized HFUS protocols and their impact on therapeutic outcomes are warranted to further consolidate its role in clinical pathways.

## Data Availability

The original contributions presented in the study are included in the article/Supplementary Material, further inquiries can be directed to the corresponding author/s.
